# Phosphoproteome of Human Glioblastoma Initiating Cells Reveals Novel Signaling Regulators Encoded by the Transcriptome

**DOI:** 10.1371/journal.pone.0043398

**Published:** 2012-08-17

**Authors:** Hiroko Kozuka-Hata, Yukiko Nasu-Nishimura, Ryo Koyama-Nasu, Hiroko Ao-Kondo, Kouhei Tsumoto, Tetsu Akiyama, Masaaki Oyama

**Affiliations:** 1 Medical Proteomics Laboratory, Institute of Medical Science, University of Tokyo, Minato-ku, Tokyo, Japan; 2 Laboratory of Molecular and Genetic Information, Institute of Molecular and Cellular Biosciences, University of Tokyo, Bunkyo-ku, Tokyo, Japan; University of Florida, United States of America

## Abstract

**Background:**

Glioblastoma is one of the most aggressive tumors with poor prognosis. Although various studies have been performed so far, there are not effective treatments for patients with glioblastoma.

**Methodology/Principal Findings:**

In order to systematically elucidate the aberrant signaling machinery activated in this malignant brain tumor, we investigated phosphoproteome dynamics of glioblastoma initiating cells using high-resolution nanoflow LC-MS/MS system in combination with SILAC technology. Through phosphopeptide enrichment by titanium dioxide beads, a total of 6,073 phosphopeptides from 2,282 phosphorylated proteins were identified based on the two peptide fragmentation methodologies of collision induced dissociation and higher-energy C-trap dissociation. The SILAC-based quantification described 516 up-regulated and 275 down-regulated phosphorylation sites upon epidermal growth factor stimulation, including the comprehensive status of the phosphorylation sites on stem cell markers such as nestin. Very intriguingly, our in-depth phosphoproteome analysis led to identification of novel phosphorylated molecules encoded by the undefined sequence regions of the human transcripts, one of which was regulated upon external stimulation in human glioblastoma initiating cells.

**Conclusions/Significance:**

Our result unveils an expanded diversity of the regulatory phosphoproteome defined by the human transcriptome.

## Introduction

Stem cells have been known to exist in each tissue of multicellular organisms and have the ability to differentiate into various cell types based on their self-renewal and differentiation potency [1]. Although the existence of cancer stem cells had been postulated for decades, there had been no experimental evidence for their presence. Recent studies demonstrated the existence of cancer stem cells in glioblastoma [2]–[6], the most aggressive brain tumors with median survival of less than 12 months after diagnosis [7], [8]. At present, the major therapies for glioblastoma are limited to radiation and anti-cancer drugs to target proliferating cells. Due to the resistance of glioblastoma stem cells to these treatments [9]–[11], however, little effect was observed for survival of patients. Therefore, in order to develop potential therapeutic strategies for the treatment of glioblastoma, functional roles of glioblastoma stem/initiating cells in tumor progression are required to be understood.

Signal transduction system transmits cellular information into nucleus in response to external stimuli via posttranslational modifications (PTMs) [12]–[14] and plays a critical role in regulating fundamental biological events such as cell growth, proliferation, and differentiation. Above all, reversible phosphorylation events are widely recognized as a central player in tumor growth. For example, the ErbB receptor family, one of the most studied receptor tyrosine kinases in vivo and in vitro, is activated by various types of ligands including epidermal growth factor (EGF), transforming growth factor alpha (TGF-α), and heregulin (HRG), leading to widespread phosphorylation of representative downstream signaling cascades such as mitogen-activated protein kinase (MAPK) and phosphoinositide 3-kinase (PI3K)/AKT signaling pathways [14]–[16] to promote various types of tumor development.

Although phosphorylation events in cancer cell signaling have largely been studied in numerous biological contexts for many years, network-wide description of each signaling dynamics is essentially needed to theoretically define signaling machinery at the system level. Recent mass spectrometry-based proteomics technology enables us to identify and quantify thousands of proteins based on shotgun strategies using SILAC, which is an *in vivo* protein labeling technique to precisely evaluate quantitative behavior of signaling molecules [17]–[19]. Through phosphopeptide enrichment by strong cation exchange (SCX) and titanium dioxide (TiO_2_) chromatography, the previous analysis quantitatively described the dynamics of phosphorylation sites in EGF-stimulated HeLa cells using high-resolution LC-MS/MS system in combination with SILAC technology, which provided a global view of cellular regulation via phosphorylation [20].

As it is well-known that amplification of EGF receptor frequently occurs in glioblastoma tumors, elevated EGF signaling is considered to make a substantial contribution to malignant character of glioblastoma stem cells. Based on the methodology developed for adherent culture of glioblastoma stem cells [21], we applied SILAC technology to primary cultured initiating cells established from glioblastoma patients and perform a global phosphoproteomics analysis in response to EGF stimulation to uncover the system-wide mechanisms for promoting brain aggressive tumorigenesis. As a result, 6,073 phosphopeptides on 2,282 human proteins were identified from glioblastoma initiating cell lysates using high-resolution nanoflow LC-MS/MS system. Characterization by gene ontology classification indicated that the two most frequent protein subgroups belonged to the terms of transcriptional activity and nucleic acid binding, which is in accordance with the previous phosphoproteome reports on human embryonic stem cells [22]. Our large-scale phosphoproteome data also demonstrated that the well-known markers of glioblastoma initiating cells and mesenchymal cells were highly phosphorylated. Very interestingly, further exploration for the human transcriptome database revealed the direct evidence that some proteins translated from novel open reading frames were also phosphorylated and regulated upon EGF stimulation in a cell-type dependent manner.

## Results

### Identification and Quantification of the Phosphoproteome in Glioblastoma Initiating Cells

**Figure 1:**
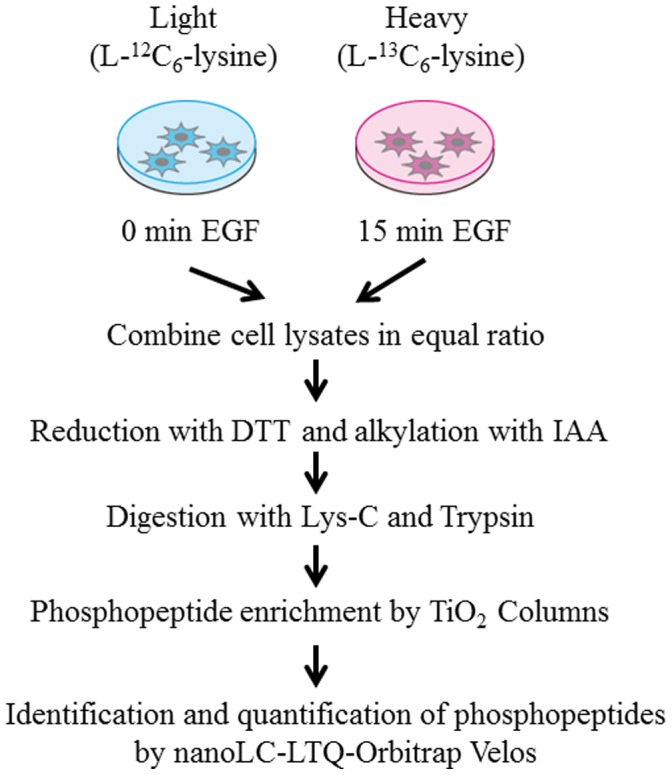
Schematic procedure for comprehensive identification and quantification of EGF-induced phosphoproteome based on SILAC technology. Two populations of glioblastoma initiating cells were grown in media supplemented with normal (L-^12^C_6_-lysine) and stable isotopes (L-^13^C_6_-lysine), respectively. Each cell population is unstimulated or stimulated with 20 ng/ml EGF for 15 min, lysed, and combined in equal ratio. Phosphopeptides enriched through TiO_2_ columns are subjected to mass spectrometric analysis.

**Figure 2:**
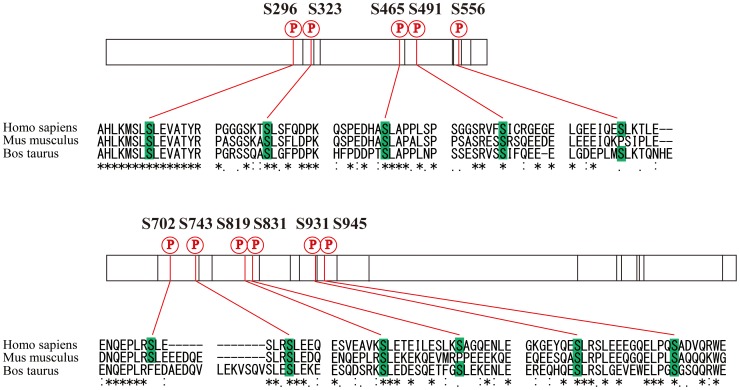
In vivo phosphorylation sites of nestin protein identified from human glioblastoma stem cells with multiple amino acid sequence alignment of Homo sapiens (NP_006608.1), Mus musculus (NP_057910.3) and Bos Taurus (NP_001193520.1). The numbers indicate the positions of the phosphorylated amino acid residues on the human sequence. The red vertical lines indicate novel phosphorylation sites, whereas the black ones represent known phosphorylation sites. The sequence alignment was performed using CLUSTALW ver. 1.83 (http://clustalw.ddbj.nig.ac.jp/top-j.html).

As amplification or mutation of EGF receptor frequently occurs in glioblastoma tumors, aberrant EGF signaling is considered to make a substantial contribution to malignant character of glioblastoma stem cells. In order to investigate EGF-induced signaling alteration at the network level, we performed global phosphoproteome analyses of SILAC-encoded glioblastoma initiating cells using high-resolution nanoflow LC-MS/MS system (Figure 1). In our shotgun phosphoproteome analyses of human glioblastoma initiating cells, a total of 60,455 redundant phosphopeptides were identified, whereas 8,424 non-phosphorylated peptides were detected with redundancy, which indicated that enrichment of phosphorylated molecules from peptide mixtures achieved high selectivity (88%) in our sample preparation. Our results revealed 6,073 non-redundant phosphopeptides derived from 2,282 proteins in total using two different peptide fragmentation methodologies of CID and HCD (Table S1). The dataset included 5,497 singly and 576 multiply phosphorylated peptides, respectively. Intriguingly, thirty-six phosphorylation sites, including eleven novel sites, were identified regarding nestin, which is a well-known marker of glioblastoma stem cells (Table 1). Many of the novel phosphorylated residues were conserved across species, which indicates functional constraint on these sites (Figure 2). A large number of phosphopeptides derived from vimentin, which is commonly regarded as a marker of mesenchymal cells, were also identified in our phosphoproteome analysis, which reflected the previous evidence that the cells undergoing epithelial to mesenchymal transition (EMT) showed the characteristic of cancer stem cells [23]. In addition, four phosphorylation sites on two phophopeptides of EGF receptor were also identified. EGF receptor signaling pathway was reported to affect on differentiation and migration of glioblastoma stem cells [24]. These results indicate that SILAC-encoded cells established in our study maintained the main characteristics of glioblastoma initiating cells.

**Table 1:** Relative quantification data on each nestin phosphorylation site by SILAC technology.

Phospho Sites^a^	Ave.	S.D.
T315	0.97	0.00
S323	1.03	0.00
S325	1.00	0.05
S465	1.35	0.00
S471	1.24	0.11
S471, S476	1.03	0.03
S548	1.24	0.02
S556	1.18	0.00
S578	0.88	0.20
S680	1.20	0.06
S702	0.96	0.00
S768	0.96	0.21
T776	1.03	0.09
S790	1.08	0.00
S820	1.02	0.17
S831	1.78	0.02
S842	1.08	0.23
T845	1.25	0.00
S905	1.15	0.05
S1016	0.94	0.20
S1347	1.05	0.16
S1577	1.11	0.29

a Evaluation of the phosphorylated sites was conductd on the basis of the threshold of above 75% phosphoRS site probabilities, which were calculated by Proteome Discover (ver. 1.3; Thermo Fisher Scientific).

**Figure 3:**
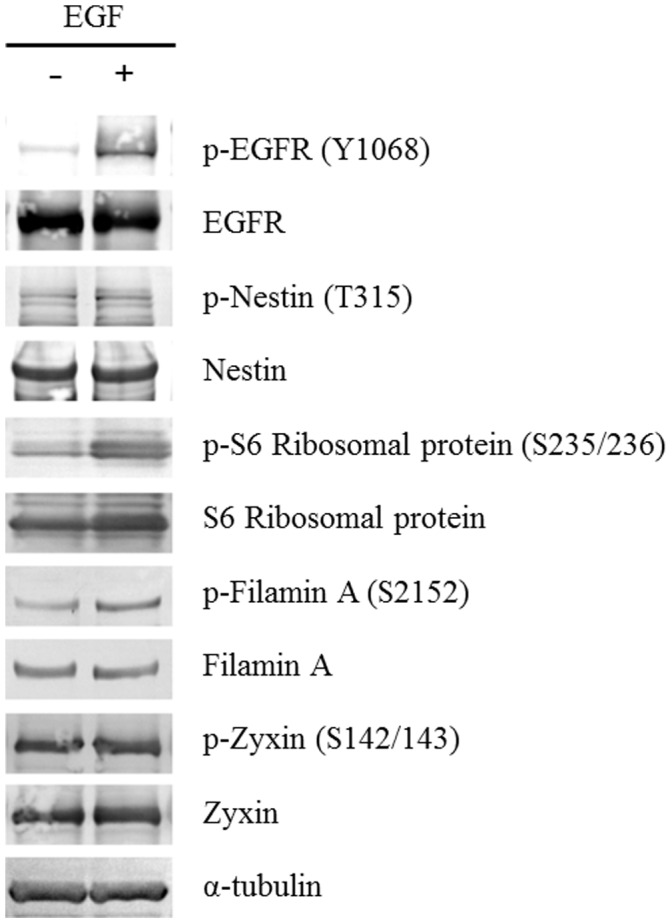
Validation of the phosphorylation status regarding the representative phosphoproteins by western blot analyses. Glioblastoma initiating cells were treated with 20 ng/ml EGF for 15 min and subjected to immunoblotting with the corresponding antibodies.

SILAC-based relative quantification of the activation fold changes revealed 516 up-regulated and 275 down-regulated phosphorylation sites which showed more than 1.5 fold change upon EGF stimulation (Table S2). The representative MS spectra of SILAC-encoded phosphopeptides derived from four phosphoproteins (nestin, 40 S ribosomal protein S6, filamin-A isoform 1 and zyxin) were shown (Figure S1) and western blot analyses using each phospho-specific antibody provided further evidence for the elevated phosphorylation of the corresponding sites of ribosomal protein S6 and filamin-A upon EGF stimulation (Figure 3). Furthermore, the reproducibility of our quantitative data from four independent experiments was also confirmed regarding representative phosphopeptides identified in all of the measurements (Table S3).

### Gene Ontology Analysis of the Glioblastoma Phosphoproteome

**Figure 4:**
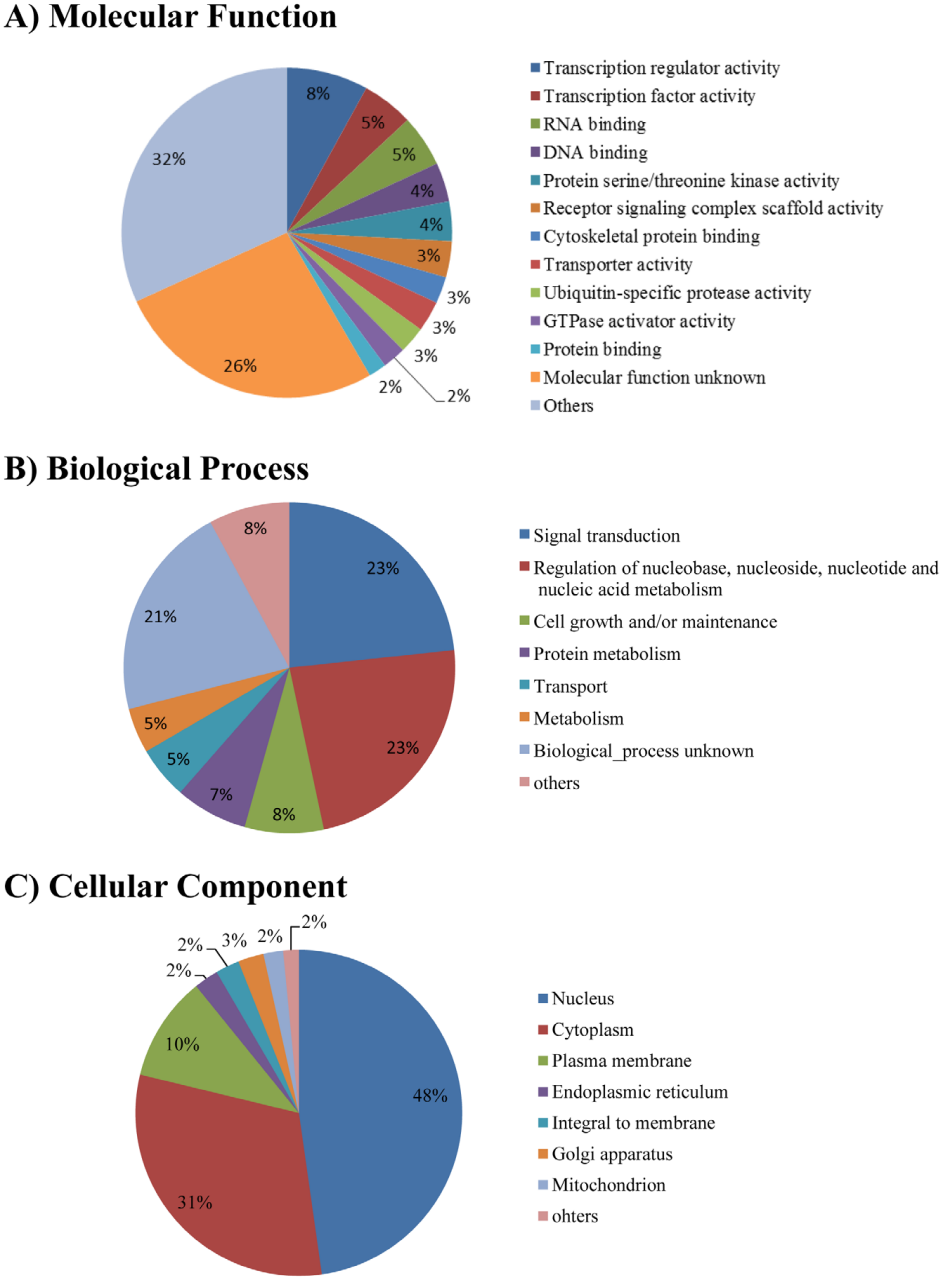
Functional classification of the glioblastoma phosphoproteome based on the Gene Ontology information in the Human Protein Reference Database (HPRD) ( http://www.hprd.org/ **).** The phosphorylated proteins identified in our analysis are classified by (A) molecular function, (B) biological process and (C) cellular component, respectively.

In order to obtain a system-level view of the glioblastoma phosphoproteome networks, all the phosphorylated proteins identified in our analyses were classified in terms of biological process, molecular function, and cellular component based on the information in the Human Protein Reference Database (HPRD) (Figure 4) [25].

According to the annotation, the largest subgroup in terms of molecular function was the factors with transcription activity, constituting 13% of the identified phosphoproteins. The group contained a variety of transcription-related factors, such as histone deacetylase, histone-lysine N-methyltransferase, and a variety of zinc finger proteins. The classification regarding cellular component showed that the phosphorylated molecules were localized in various organelles, especially in the nucleus and cytoplasm (48% and 31% of all the identified proteins, respectively). The classification by biological process showed that the molecules belonging to signal transduction (23%), cell regulation of nucleobase, nucleoside, nucleotide and nucleic acid metabolism (23%), and cell growth and/or maintenance (8%) were the major populations. For further dissection, Database for Annotation, Visualization and Integrated Discovery (DAVID) [26] software was used to classify the phosphoproteome data into the related signaling pathways (Table 2). As a result, they were assigned to diverse signaling pathways including Notch and EGF receptor signaling pathways. Notch signaling has been found to be associated with various types of tumors including gliomas and play crucial roles in regulating self-renewal and determining the fate of neural stem cells, while EGF receptor signaling is known to be required for maintaining stemness of glioblastoma [27]. Regarding the ErbB signaling pathway, some phosphorylation sites on GRB2-associated binding protein 1 (GAB1), SHC (Src homology 2 domain containing) transforming protein 1 (SHC1) and v-raf-1 murine leukemia viral oncogene homolog 1(RAF1) were found to be up-regulated upon EGF stimulation in our phosphoproteome analyses (Table S4). Very interestingly, ribosomal protein S6 (RPS6) in the mTOR signaling was observed to be highly phosphorylated upon this input in human glioblastoma initiating cells.

**Table 2:** List of the phosphorylated molecules involved in representative glioblastoma-related signaling pathways classified by DAVID^a^.

Pathway	Protein name	Gene symbol
Notch signaling pathway	ADAM metallopeptidase domain 17	ADAM17
	E1A binding protein p300	EP300
	SNW domain containing 1	SNW1
	corepressor interacting with RBPJ	CIR1
	deltex 3-like (Drosophila)	DTX3L
	dishevelled, dsh homolog 3 (Drosophila)	DVL3
	histone deacetylase 1	HDAC1
	histone deacetylase 2	HDAC2
	mastermind-like 2 (Drosophila)	MAML2
	nuclear receptor co-repressor 2	NCOR2
	numb homolog (Drosophila)-like	NUMBL
	presenilin 1	PSEN1
	presenilin 2 (Alzheimer disease 4)	PSEN2
ErbB signaling pathway	BCL2-associated agonist of cell death	BAD
	Cas-Br-M (murine) ecotropic retroviral transforming sequence	CBL
	Cas-Br-M (murine) ecotropic retroviral transforming sequence b	CBLB
	GRB2-associated binding protein 1	GAB1
	NCK adaptor protein 1	NCK1
	NCK adaptor protein 2	NCK2
	PTK2 protein tyrosine kinase 2	PTK2
	SHC (Src homology 2 domain containing) transforming protein 1	SHC1
	SHC (Src homology 2 domain containing) transforming protein 3	SHC3
	c-abl oncogene 1, receptor tyrosine kinase	ABL1
	calcium/calmodulin-dependent protein kinase II delta	CAMK2D
	calcium/calmodulin-dependent protein kinase II gamma	CAMK2G
	cyclin-dependent kinase inhibitor 1B (p27, Kip1)	CDKN1B
	epidermal growth factor receptor (erythroblastic leukemia viral (v-erb-b) oncogene homolog, avian)	EGFR
	eukaryotic translation initiation factor 4E binding protein 1	EIF4EBP1
	glycogen synthase kinase 3 beta	GSK3B
	hypothetical LOC100271831; mitogen-activated protein kinase 3	MAPK3
	jun oncogene	JUN
	mitogen-activated protein kinase 1	MAPK1
	mitogen-activated protein kinase kinase 2 pseudogene; mitogen-activated protein kinase kinase 2	MAP2K2
	mitogen-activated protein kinase kinase 7	MAP2K7
	p21 protein (Cdc42/Rac)-activated kinase 2	PAK2
	p21 protein (Cdc42/Rac)-activated kinase 4	PAK4
	protein kinase C, alpha	PRKCA
	protein kinase C, beta	PRKCB
	signal transducer and activator of transcription 5B	STAT5B
	v-abl Abelson murine leukemia viral oncogene homolog 2 (arg, Abelson-related gene)	ABL2
	v-akt murine thymoma viral oncogene homolog 1	AKT1
	v-akt murine thymoma viral oncogene homolog 2	AKT2
	v-akt murine thymoma viral oncogene homolog 3 (protein kinase B, gamma)	AKT3
	v-crk sarcoma virus CT10 oncogene homolog (avian)	CRK
	v-crk sarcoma virus CT10 oncogene homolog (avian)-like	CRKL
	v-raf murine sarcoma 3611 viral oncogene homolog	ARAF
	v-raf murine sarcoma viral oncogene homolog B1	BRAF
	v-raf-1 murine leukemia viral oncogene homolog 1	RAF1
	v-src sarcoma (Schmidt-Ruppin A-2) viral oncogene homolog (avian)	SRC
mTOR signaling pathway	3-phosphoinositide dependent protein kinase-1	PDPK1
	RPTOR independent companion of MTOR, complex 2	RICTOR
	eukaryotic translation initiation factor 4E binding protein 1	EIF4EBP1
	eukaryotic translation initiation factor 4E family member 2	EIF4E2
	hypothetical LOC100271831; mitogen-activated protein kinase 3	MAPK3
	mitogen-activated protein kinase 1	MAPK1
	protein kinase, AMP-activated, alpha 1 catalytic subunit	PRKAA1
	regulatory associated protein of MTOR, complex 1	RPTOR
	ribosomal protein S6 kinase, 90 kDa, polypeptide 1	RPS6KA1
	ribosomal protein S6 kinase, 90 kDa, polypeptide 3	RPS6KA3
	ribosomal protein S6 pseudogene 25; ribosomal protein S6; ribosomal protein S6 pseudogene 1	RPS6
	serine/threonine kinase 11	STK11
	similar to eukaryotic translation initiation factor 4H; eukaryotic translation initiation factor 4B	EIF4B
	tuberous sclerosis 1	TSC1
	tuberous sclerosis 2	TSC2
	v-akt murine thymoma viral oncogene homolog 1	AKT1
	v-akt murine thymoma viral oncogene homolog 2	AKT2
	v-akt murine thymoma viral oncogene homolog 3 (protein kinase B, gamma)	AKT3
	v-raf murine sarcoma viral oncogene homolog B1	BRAF
Glioma	SHC (Src homology 2 domain containing) transforming protein 1	SHC1
	SHC (Src homology 2 domain containing) transforming protein 3	SHC3
	calcium/calmodulin-dependent protein kinase II delta	CAMK2D
	calcium/calmodulin-dependent protein kinase II gamma	CAMK2G
	epidermal growth factor receptor (erythroblastic leukemia viral (v-erb-b) oncogene homolog, avian)	EGFR
	hypothetical LOC100271831; mitogen-activated protein kinase 3	MAPK3
	mitogen-activated protein kinase 1	MAPK1
	mitogen-activated protein kinase kinase 2 pseudogene; mitogen-activated protein kinase kinase 2	MAP2K2
	platelet-derived growth factor receptor, alpha polypeptide	PDGFRA
	protein kinase C, alpha	PRKCA
	protein kinase C, beta	PRKCB
	retinoblastoma 1	RB1
	tumor protein p53	TP53
	v-akt murine thymoma viral oncogene homolog 1	AKT1
	v-akt murine thymoma viral oncogene homolog 2	AKT2
	v-akt murine thymoma viral oncogene homolog 3 (protein kinase B, gamma)	AKT3
	v-raf murine sarcoma 3611 viral oncogene homolog	ARAF
	v-raf murine sarcoma viral oncogene homolog B1	BRAF
	v-raf-1 murine leukemia viral oncogene homolog 1	RAF1
Cell Cycle	CHK1 checkpoint homolog (S. pombe)	CHEK1
	E1A binding protein p300	EP300
	MAD1 mitotic arrest deficient-like 1 (yeast)	MAD1L1
	S-phase kinase-associated protein 1	SKP1
	S-phase kinase-associated protein 2 (p45)	SKP2
	SMAD family member 2	SMAD2
	WEE1 homolog (S. pombe)	WEE1
	anaphase promoting complex subunit 1; similar to anaphase promoting complex subunit 1	ANAPC1
	anaphase promoting complex subunit 4	ANAPC4
	ataxia telangiectasia and Rad3 related; similar to ataxia telangiectasia and Rad3 related protein	ATR
	budding uninhibited by benzimidazoles 1 homolog (yeast)	BUB1
	c-abl oncogene 1, receptor tyrosine kinase	ABL1
	cell division cycle 16 homolog (S. cerevisiae)	CDC16
	cell division cycle 2, G1 to S and G2 to M	CDK1
	cell division cycle 23 homolog (S. cerevisiae)	CDC23
	cell division cycle 25 homolog B (S. pombe)	CDC25B
	cell division cycle 26 homolog (S. cerevisiae); cell division cycle 26 homolog (S. cerevisiae) pseudogene	CDC26
	cyclin-dependent kinase 2	CDK2
	cyclin-dependent kinase 7	CDK7
	cyclin-dependent kinase inhibitor 1B (p27, Kip1)	CDKN1B
	glycogen synthase kinase 3 beta	GSK3B
	histone deacetylase 1	HDAC1
	histone deacetylase 2	HDAC2
	minichromosome maintenance complex component 2	MCM2
	minichromosome maintenance complex component 3	MCM3
	minichromosome maintenance complex component 6	MCM6
	pituitary tumor-transforming 1; pituitary tumor-transforming 2	PTTG2
	protein kinase CHK2-like; CHK2 checkpoint homolog (S. pombe); similar to hCG1983233	CHEK2
	retinoblastoma 1	RB1
	similar to 14-3-3 protein epsilon (14-3-3E) (Mitochondrial import stimulation factor L subunit) (MSF L);	YWHAE
	tyrosine 3-monooxygenase/tryptophan 5-monooxygenase activation protein, epsilon polypeptide	
	similar to Serine-protein kinase ATM (Ataxia telangiectasia mutated) (A-T, mutated); ataxia telangiectasia mutated	ATM
	stromal antigen 2	STAG2
	structural maintenance of chromosomes 3	SMC3
	tumor protein p53	TP53
	tyrosine 3-monooxygenase/tryptophan 5-monooxygenase activation protein, zeta polypeptide	YWHAZ

a http://david.abcc.ncifcrf.gov/home.jsp.

### Exploration for Novel EGF Effectors Encoded by the Human Transcriptome

In order to search for the novel phosphopeptides that were not derived from known protein sequences, we further performed searching against the human RNA database. As a result, three novel phosphopeptides were additionally identified from the nucleotide sequences presumed as non-coding regions (Table 3). Two phosphopeptides were derived from the regions different from already defined protein-coding sequences on mRNAs, whereas the other was from the regions on non-coding RNAs (Table S5). The surrounding amino acids of the novel phosphorylated residue identified from nuclear protein localization 4 homolog (S. cerevisiae) were well conserved across species, which indicates functional constraint on the phosphorylation site (Figure S2). Regarding the novel effector encoded by supervillin-like (LOC645954), we also analyzed sequence similarity with the already known supervillin protein by aligning the corresponding amino acid sequences and found that these sequences including the novel phosphorylation residue showed high similarity (Figure S3A). The phosphorylation level of this novel molecule was increased by 4-fold on average in response to EGF stimulation (Table S5), whereas, very intriguingly, its phosphorylation level was not altered upon EGF treatment in other human cancer cells (Figure S3B). Our results indicate that this novel EGF effector identified from glioblastoma initiating cells behaved in a cell-type dependent manner.

**Table 3:** List of the novel phosphorylated peptides extracted from NCBI Refseq human RNA database.

Accession	Definition	Identified Peptide Sequence^a^
XR_017086.3	PREDICTED: Homo sapiens supervillin-like (LOC645954), miscRNA	GLApSPTAITPVASAICGK
NM_020134.3	Homo sapiens dihydropyrimidinase-like 5 (DPYSL5), mRNA	SIpSEENMLANSASVR
NM_017921.2	Homo sapiens nuclear protein localization 4 homolog (S. cerevisiae) (NPLOC4), mRNA	GSpSPEAGAAAMAESIIIR

a Evaluation of the phosphorylated sites was conductd on the basis of the threshold of above 75% phosphoRS site probabilities, which were calculated by Proteome Discover (ver. 1.3; Thermo Fisher Scientific). The phosphorylated amino acid residues were indicated by pS, pT and pY, respectively.

## Discussion

Cancer stem/initiating cells are known to have the ability of self-renewal, multi-lineage differentiation and proliferation. Due to their characteristics of chemotherapy and radiotherapy resistance, current conventional treatments have limited efficacy in curative therapies for each cancer. Especially, glioblastoma is recognized as the most aggressive tumors with poor prognosis. In order to develop potential therapeutic targets for glioblastoma initiating cells, it is essential to understand the molecular mechanisms underlying aberrant signaling behavior in these cells. As reversible phosphorylation-dephosphorylation signaling events in cancer cells are known to play a pivotal role in transmitting signals from receptors to the nucleus, we applied high-resolution mass spectrometry-based proteomics technology to comprehensively unravel global phosphoproteome dynamics in glioblastoma initiating cells.

There are some recent reports in which proteomics approaches were used to characterize molecular mechanisms of stem cells [22], [28], [29] and cancer stem cells [30], [31]. The tyrosine-phosphoproteome in the cells with highly expressed EGF receptor variant III (EGFRvIII), the most common variant of the EGF receptor observed in glioblastoma multiforme, were quantitatively analyzed upon EGF stimulation [32]. Quantitative phosphorylation events focused on signal transducer and activator of transcription 3 (STAT3)/interleukin 6 (IL6)/hypoxia-inducible factor 2α (HIF2α) autocrine loop were also reported regarding glioblastoma stem cells [33]. The strategies for quantification of activation fold change in the studies above were based on in vitro labeling technologies, leading to generation of less accurate quantitative information on activation change than in vivo labeling such as SILAC.

Based on the two different peptide fragmentation methodologies of CID and HCD, we detected 6,073 phosphopeptides from 2,282 phosphoproteins in total, including various factors mainly related to signal transduction and transcriptional regulation. Among the proteins identified, 635 phosphorylated molecules including GAB1, SHC1, EIF4EBP1, RAF1 and RPS6 in the context of ErbB and mTOR signaling showed more than 1.5 fold change upon EGF stimulation (Table S4). The phosphorylated status of EIF4EBP1 and RPS6 is widely known to facilitate protein translation and the related mTOR signaling pathway is closely involved in regulating stem cell proliferation [34], [35]. Therefore, these results suggest that EGF might play a part in regulating glioblastoma initiating cell proliferation.

Our phosphoproteome data also indicated that some marker molecules for glioblastoma initiating cells, such as nestin and vimentin, were found to be highly phosphorylated with many novel phosphorylation sites in addition to previously reported ones on these key molecules. These results indicate that SILAC-encoded glioblastoma initiating cells established in our study showed the characteristics of stemness even after repeated subculture.

The International Human Genome Sequencing Consortium has indicated that there are only ∼20,000 protein-coding genes in the human genome [36]. The number of genes does not significantly differ between human and many other species, inferring that non-coding RNAs might be responsible for the complexity of cancer-related cellular signaling systems. Although the biological functions of short RNAs such as microRNAs remain largely unknown, recent studies regarding human embryonic stem cells showed that microRNA-145 directly targeted pluripotency factors such as OCT4, SOX2 and KLF4 and induced cell differentiation [37]. There is also another report in which screening for autoantibody derived from prostate cancer tissues showed that, among the 22 peptides used as a detector, 18 were generated from the untranslated regions of the transcripts [38].

Thus we investigated whether protein molecules encoded by previously undefined regions in the human transcriptome were involved in the phosphoproteome dynamics in EGF signaling of human glioblastoma initiating cells. Indeed, our shotgun phosphoproteome analysis based on high-resolution mass spectrometry led to identification of three phosphorylated peptides defined by novel coding regions on the human transcripts. Very interestingly, the phosphorylation level on one novel peptide encoded by supervillin-like (LOC645954) was altered upon external EGF stimulation in a cell-type dependent manner. This finding provided the first direct evidence that novel phosphorylated molecules encoded by the transcripts acted as signaling effectors in response to external stimulation, which indicates the possibility of the involvement of such previously unrecognized factors in tumorigenic potential of cancer stem-like cells. Further comprehensive analyses of phosphoproteome dynamics in many types of cancer stem-like cells will contribute to systematic definition of the core signaling machinery responsible for cancer progression.

## Materials and Methods

### Cell Culture

Glioblastoma initiating cells were originally established from the glioblastoma brain tissues in the University of Tokyo Hospital based on the written informed consent to undertake genetic and molecular analyses from the patients, which was approved by the Research Ethics Committee at the Institute of Medical Science, University of Tokyo. Among the cell populations established from the brain tissues provided by each patient, a representative one was selected on the basis of the CD15 (SSEA-1) expression level. CD15 (SSEA-1) is one of the most reliable markers for glioblastoma stem cell enrichment and the fluorescence-activated cell sorting (FACS) analysis showed that the CD15-positive rate of the selected glioblastoma initiating cell line was 12% [39]. The cells were cultured in Dulbecco’s modified Eagle’s medium: Nutrient Mixture F-12 (DMEM/F12) media (Pierce) with 2% B27 supplement minus vitamin A, 20 ng/ml EGF (Upstate Biotechnology) and 20 ng/ml fibroblast growth factor (FGF) (Roche) as previously described [21] and then applied to SILAC media (labeled with either normal L-lysine or ^13^C_6_ L-lysine, respectively). The incorporation of stable isotopes into cellular proteins was validated by MS measurement of some representative proteins including α-tubulin.

### Protein Digestion

The same media without B27 supplement, EGF and FGF were used for starvation and then treated with 20 ng/ml EGF for 0 min (normal L-lysine) or 15 min (^13^C_6_ L-lysine), respectively. The cells were washed three times with PBS, harvested, and suspended in 8 M urea containing PhosSTOP (Roche Diagnostics) and Benzonase (Novagen). After the mixture was kept left on ice for 1 h, cellular debris was pelleted by centrifugation at 15,000 rpm for 30 min. Each lysate was diluted two hundred fold, quantified using BCA Protein Assay Kit (Thermo Scientific) and mixed in equal ratio.

The proteins were reduced with 1 mM ditiothreitol (DTT) for 90 min, and subsequently alkylated with 5.5 mM iodoacetamide (IAA) for 30 min. After digestion with Lysyl Endopeptidase (Lys-C) (1∶50 w/w) (Wako) at 37°C for 3 h, the resulting peptide mixtures were diluted with 10 mM Tris-HCl (pH 8.2) to achieve final concentration below 2 M Urea and subsequently digested with modified trypsin (1∶50 w/w) (Sequencing grade, Promega) at 37°C for 3 h. The equal amount of trypsin was then added once more for overnight digestion.

### Sample Preparation for Mass Spectrometry

Phosphopeptides were enriched by Titansphere Phos-TiO Kit (GL Sciences) according to the manufacturer’s instructions. In short, after equilibration of the column, the peptide mixtures from glioblastoma initiating cell lysates were applied to Spin Tip, mixed with the buffer containing 2-hydroxypropanoic acid and centrifuged. After the column was washed, captured peptides were eluted with 5% ammonium solution and 5% pyrrolidine solution, successively. The enriched phosphopeptide solutions were acidified by 10% TFA, desalted by ZipTip C_18_ (Millipore) and centrifuged by vacuum concentrator.

### Mass Spectrometric Analysis

Shotgun proteomic analyses of the Titansphere eluates were performed by a linear ion trap-orbitrap mass spectrometer (LTQ-Orbitrap Velos, Thermo Fisher Scientific) coupled with nanoflow LC system (Dina-2A, KYA Technologies). Peptides were injected into 75 µm reversed-phase C_18_ column at a flow rate of 10 µl/min and eluted with a linear gradient of solvent A (2% acetonitrile and 0.1% formic acid in H_2_O) to solvent B (40% acetonitrile and 0.1% formic acid in H_2_O) at 300 nl/min. Peptides were sequentially sprayed from nanoelectrospray ion source (KYA Technologies) and analyzed by collision induced dissociation (CID) and higher-energy C-trap dissociation (HCD) [40], respectively. The analyses were carried out in data dependent mode, switching automatically between MS and MS/MS acquisition. For CID analyses, full-scan MS spectra (from m/z 380 to 2,000) were acquired in the orbitrap with resolution of 100,000 at m/z 400 after ion count accumulation to the target value of 1,000,000. The 20 most intense ions at a threshold above 2,000 were fragmented in the linear ion trap with normalized collision energy of 35% for activation time of 10 ms. For HCD analyses, full-scan MS spectra (from m/z 380 to 2,000) were acquired in the orbitrap with resolution of 30,000 at m/z 400 after accumulation to the target value of 1,000,000. The 10 most intense ions at a threshold above 5,000 were fragmented in the orbitrap with normalized collision energy of 35% for activation time of 0.1 ms. The orbitrap analyzer was operated with the “lock mass” option to perform shotgun detection with high accuracy [41].

### Protein Identification and Quantification

Protein identification was conducted by searching MS and MS/MS data against the RefSeq (National Center for Biotechnology Information) human protein database (32,968 protein sequences as of Sep 12, 2011) and human RNA database (42,646 RNA sequences as of Sep 12, 2011) by Mascot ver. 2.3.02 (Matrix Science). Carbamidomethylation of cysteine residues was set as fixed modifications, whereas methionine oxidation, protein N-terminal acetylation, pyro-glutamination for N-terminal glutamine, phosphorylation (Ser, Thr, and Tyr) and stable isotopes of Lys 6 were set as variable modifications. A maximum of two missed cleavages was allowed in our database search. The tolerance for mass deviation fragmented by CID and HCD was set to 3 parts per million (ppm) for peptide masses and 0.8 Da (CID)/0.01 Da (HCD) for MS/MS peaks, respectively. In the process of peptide identification, we conducted forward and reverse database searching by Mascot and applied a filter to satisfy a false positive rate lower than 1%. Regarding the proteins supported by a single peptide identification, we applied an additional criterion (E value <0.01) to evaluate their identification in a stringent manner. Determination of phosphorylated sites on the peptides and quantification of their fold change were performed using Proteome Discoverer ver 1.3 (Thermo Fisher Scientific).

### Western Blot Analysis

In order to validate the mass spectrometry data, the proteins were separated on SDS-PAGE and change of the phosphorylation level was analyzed using phospho-specific antibodies. The following antibodies were used as probes at 1∶500 dilution: phospho-EGFR (3777; Cell Signaling Technology), EGFR(4267; Cell Signaling Technology), phospho-nestin (sc-33879; Santa Cruz Biotechnology), nestin (MAB1259; R&D Systems), phospho-S6 ribosomal protein (4858; Cell Signaling Technology), S6 ribosomal protein (2217; Cell Signaling Technology), phospho-filamin A (4761; Cell Signaling Technology), filamin A (4762; Cell Signaling Technology), phospho-zyxin (8467; Cell Signaling Technology), zyxin (3553; Cell Signaling Technology), and alpha-tubulin (CP06; Calbiochem). Alpha-tubulin was analyzed as a protein loading control.

## Supporting Information

Figure S1 **Representative mass spectra of the phosphopeptides from (A) nestin (LQpTPGGGSK), (B) 40 S ribosomal protein S6 (RLpSpSLRApSTSK), (C) filamin-A isoform 1 (RAPpSVANVGSHCDLSLK) and (D) zyxin (VSpSIDLEIDSLSSLLDDMTK).** SILAC pairs were used to quantify fold changes upon EGF treatment.
(TIF)

Figure S2 **Multiple amino acid sequence alignment of the surrounding sequences of the novel phosphorylated residue identified from nuclear protein localization 4 homolog (S. cerevisiae).** The yellow box represents the peptide sequence identified in our phosphoproteome analysis, whereas the green one indicates the position of the novel phosphorylated amino acid residue. Each sequence was translated from the corresponding mRNA sequence as indicated below. Homo sapiens: NM_017921.2, Mus musculus: NM_199469.2, Bos Taurus: NM_001192189.1.
(TIF)

Figure S3 **Characterization of the novel phophorylated molecule encoded by supervillin-like (LOC645954).** (A) Multiple amino acid sequence alignment of the novel phosphorylated protein encoded by supervillin-like (LOC645954) (XR_017086.3) and supervillin isoform 1 (NP_003165.2). The yellow box represents the peptide sequence identified in our phosphoproteome analysis, whereas the green one indicates the position of the novel phosphorylated amino acid residue. (B) Mass spectra of the novel phosphopeptide (GLApSPTAITPVASAICGK) encoded by supervillin-like (LOC645954) in HeLa-derived cells and glioblastoma initiating cells upon EGF stimulation.
(TIF)

Table S1 **Detailed information on the phosphopeptides identified from NCBI RefSeq human protein database.**
(XLSX)

Table S2 **Quantitative information on the EGF-regulated phosphopeptides identified from NCBI RefSeq human protein database.**
(XLSX)

Table S3 **Comparison of relative quantification data on representative phosphopeptides from four independent SILAC experiments.**
(XLSX)

Table S4 **EGF-stimulated fold changes concerning phosphorylated molecules involved in representative glioblastoma-related signaling pathways classified by DAVID^a^.**
(XLSX)

Table S5 **Detailed information on the novel phosphopeptides identified from NCBI RefSeq human mRNA database.**
(XLSX)
