# Supplementary figures and images for: Phosphoproteome of Human Glioblastoma Initiating Cells Reveals Novel Signaling Regulators Encoded by the Transcriptome
Source: PLoS One. 2012 Aug 17;7(8):e43398. doi: 10.1371/journal.pone.0043398 (PMC3422224; doi:10.1371/journal.pone.0043398)

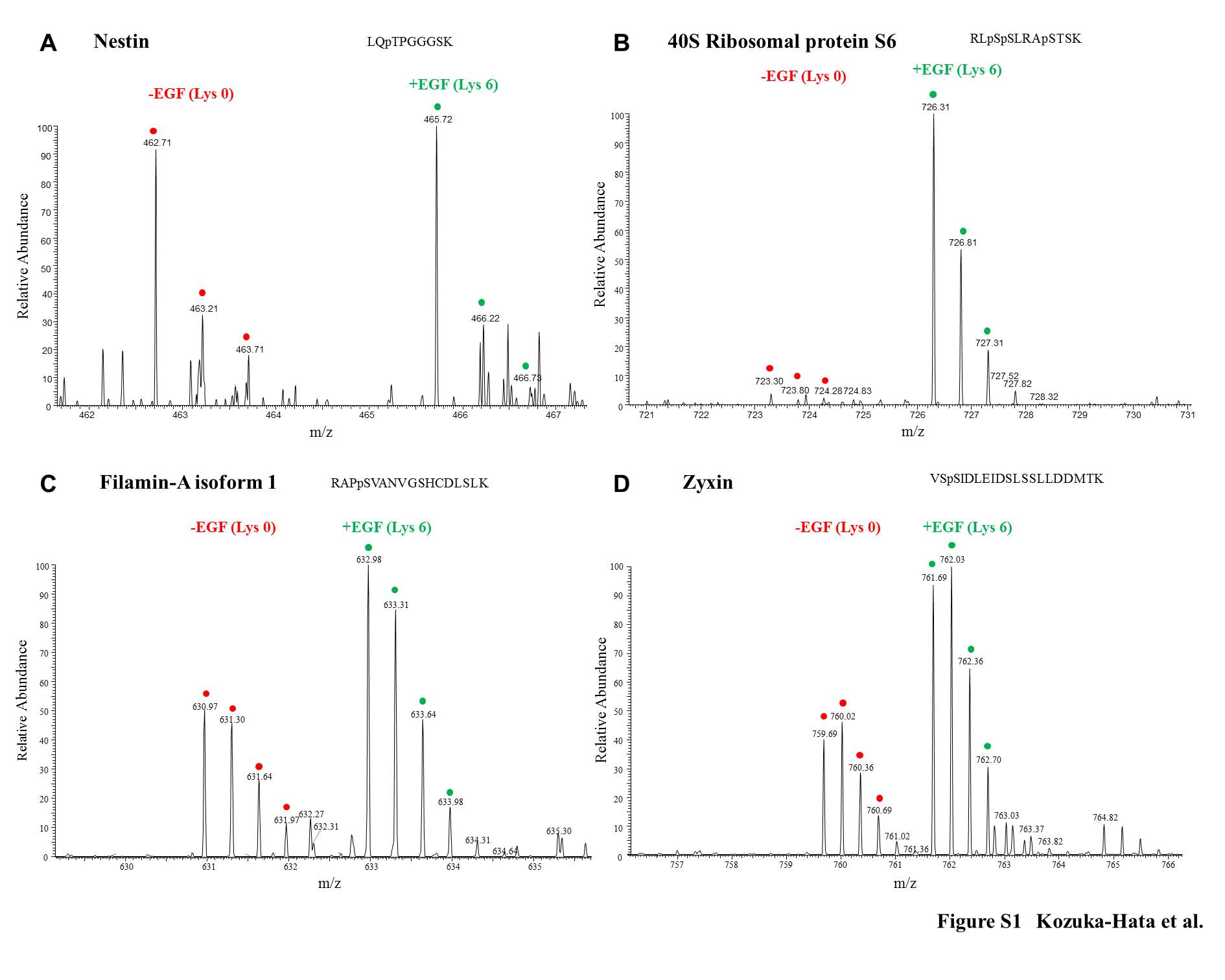

Supplement: Figure S1 — Representative mass spectra of the phosphopeptides from (A) nestin (LQpTPGGGSK), (B) 40 S ribosomal protein S6 (RLpSpSLRApSTSK), (C) filamin-A isoform 1 (RAPpSVANVGSHCDLSLK) and (D) zyxin (VSpSIDLEIDSLSSLLDDMTK). SILAC pairs were used to quantify fold changes upon EGF treatment. (TIF) [file pone.0043398.s001.tif]

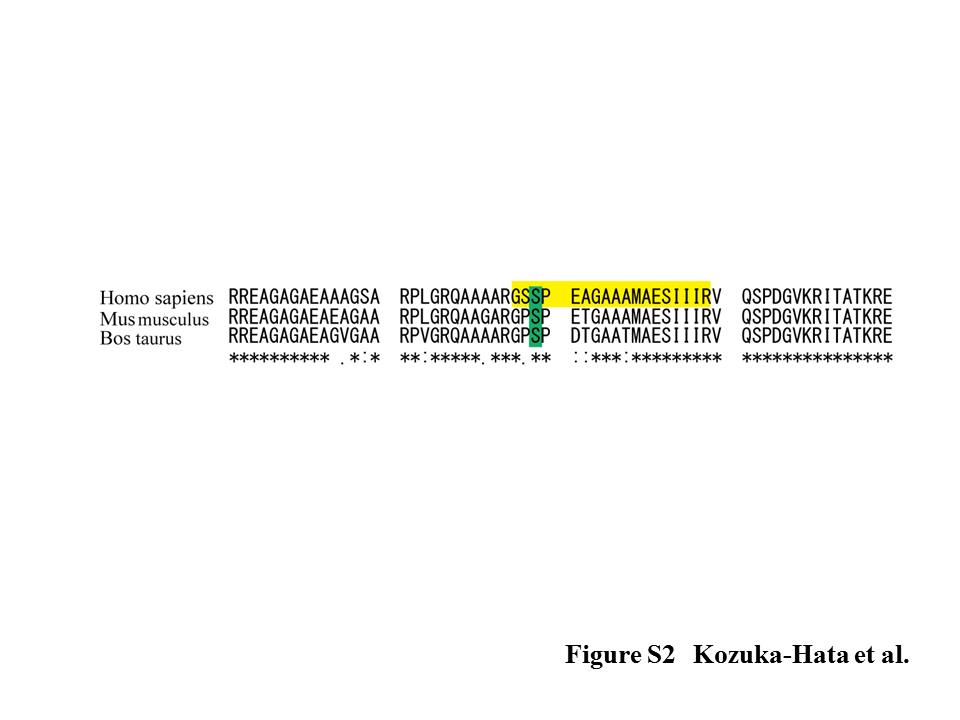

Supplement: Figure S2 — Multiple amino acid sequence alignment of the surrounding sequences of the novel phosphorylated residue identified from nuclear protein localization 4 homolog (S. cerevisiae). The yellow box represents the peptide sequence identified in our phosphoproteome analysis, whereas the green one indicates the position of the novel phosphorylated amino acid residue. Each sequence was translated from the corresponding mRNA sequence as indicated below. Homo sapiens: NM_017921.2, Mus musculus: NM_199469.2, Bos Taurus: NM_001192189.1. (TIF) [file pone.0043398.s002.tif]

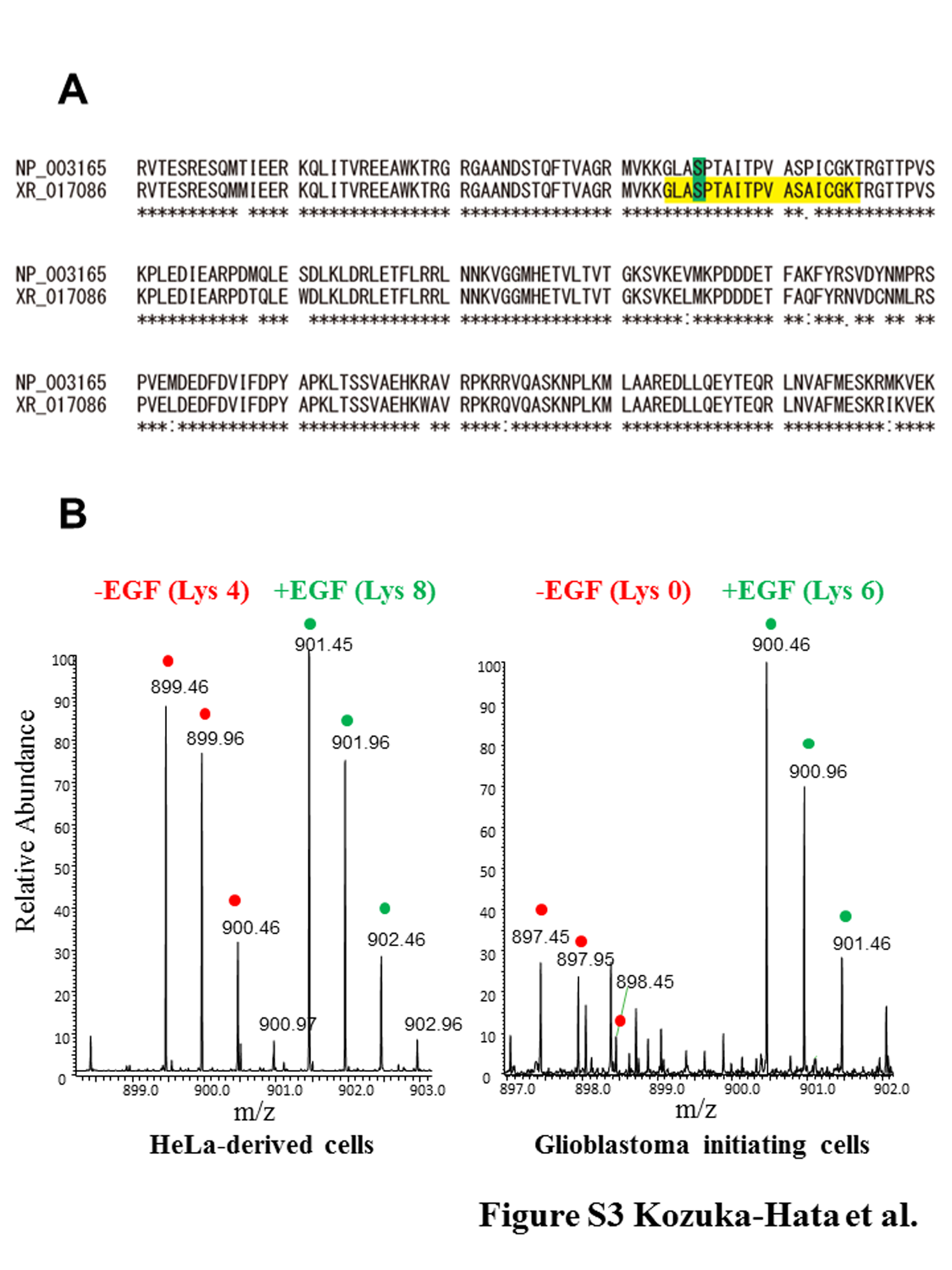

Supplement: Figure S3 — Characterization of the novel phophorylated molecule encoded by supervillin-like (LOC645954). (A) Multiple amino acid sequence alignment of the novel phosphorylated protein encoded by supervillin-like (LOC645954) (XR_017086.3) and supervillin isoform 1 (NP_003165.2). The yellow box represents the peptide sequence identified in our phosphoproteome analysis, whereas the green one indicates the position of the novel phosphorylated amino acid residue. (B) Mass spectra of the novel phosphopeptide (GLApSPTAITPVASAICGK) encoded by supervillin-like (LOC645954) in HeLa-derived cells and glioblastoma initiating cells upon EGF stimulation. (TIF) [file pone.0043398.s003.tif]
